# Comprehensive Analysis of the Expression and Prognosis for Tripartite Motif-Containing Genes in Breast Cancer

**DOI:** 10.3389/fgene.2022.876325

**Published:** 2022-07-19

**Authors:** Lvwen Ning, Qin Huo, Ni Xie

**Affiliations:** Biobank, Shenzhen Second People’s Hospital, First Affiliated Hospital of Shenzhen University, Health Science Center, Shenzhen University, Shenzhen, China

**Keywords:** TRIM family, breast cancer, pronositic biomarker, bioinformatics, TRIM45

## Abstract

Tripartite motif-containing genes (TRIMs), with a ubiquitin ligase’s function, play critical roles in antitumor immunity by activating tumor-specific immune responses and stimulating tumor proliferation, thus affecting patient outcomes. However, the expression pattern and prognostic values of TRIMs in breast cancer (BC) are not well clarified. In this study, several datasets and software were integrated to perform a comprehensive analysis of the expression pattern in TRIMs and investigate their prognosis values in BC. We found that *TRIM59/46* were significantly upregulated and *TRIM66/52-AS1/68/7/2/9/29* were decreased in BC and validated them using an independent cohort. The expression of numerous TRIMs are significantly correlated with BC molecular subtypes, but not with tumor stages or patient age at diagnosis. Higher expression of *TRIM3*/*14*/*69*/*45* and lower expressions of *TRIM68*/*2* were associated with better overall survival in BC using the Kaplan–Meier analysis. The multivariate Cox proportional hazards model identified *TRIM45* as an independent prognostic marker. Further analysis of single-cell RNA-seq data revealed that most TRIMs are also expressed in nontumor cells. Higher expression of some TRIMs in the immune or stromal cells suggests an important role of TRIMs in the BC microenvironment. Functional enrichment of the co-expression genes indicates that they may be involved in muscle contraction and interferon-gamma signaling pathways. In brief, through the analysis, we provided several TRIMs that may contribute to the tumor progression and *TRIM45* as a potential new prognostic biomarker for BC.

## 1 Introduction

Breast cancer (BC) is a complex disease with a high incidence worldwide, especially in women (Siegel et al., 2021). An approximate of 2.3 million new cases were diagnosed in 2020 (Sung et al., 2021). Based on the gene expression pattern, BC is classified into five intrinsic subtypes: luminal A, luminal B, HER2+, basal-like, and normal-like (Sørlie et al., 2003). Different subtypes usually require different treatment strategies (Rouzier et al., 2005). Although great progress has been made recently in molecular diagnostic and neoadjuvant chemotherapy (Waks and Winer, 2019), there are still ∼680,000 BC patients who die each year (Sung et al., 2021). Therefore, discovering new biomarkers for stratified BC patients and new therapeutic targets is urgent.

The tripartite motif-containing genes (TRIMs) are involved in many cellular functions by acting as E3 ubiquitin ligases (Ozato et al., 2008; Lv et al., 2017; van Gent et al., 2018). As a result, the dysregulation of TRIMs results in a lot of diseases (Jefferies et al., 2011; Borlepawar et al., 2019; Kumarasinghe et al., 2021), including various types of cancer (Cambiaghi et al., 2012; Crawford et al., 2018; Jaworska et al., 2020). More than 80 members from this conserved protein superfamily are reported in humans (Sardiello et al., 2008). Several TRIMs play an oncogenic role in cancers, while some exert tumor suppressor functions (Venuto and Merla, 2019). Earlier, our group found that rare variants in *TRIM31* have contributed to the genetic susceptibility of nasopharyngeal carcinoma (NPC), and higher TRIM31 expression is associated with poor overall survival (OS) of NPC (Ning et al., 2020). We recently reported that TRIM58 can promote drug resistance in BC (Wang et al., 2022). TRIM28 (Wei et al., 2016), TRIM44 (Kawabata et al., 2017), and TRIM59 (Tan et al., 2018) were reported to play an oncogene role, and TRIM16 (Bell et al., 2013) was a tumor suppressor gene in BC. The important role of TRIMs in BC suggests that some TRIMs may have a prognostic value in BC. Higher expression of TRIM44 (Kawabata et al., 2017) and lower expression of TRIM13 (Chen et al., 2019)/TRIM21 (Zhou et al., 2018) are poor prognostic factors in BC.

Although the expression levels of some TRIMs have been reported to have prognostic values, the expression pattern of TRIMs and their prognostic values are not comprehensively clarified in BC. The functional study of this family is still limited. In this study, by integrating several datasets, we explored the expression profile of all TRIMs in BC, prioritized several not well-studied TRIMs that may contribute to BC tumorigenesis, and identified *TRIM45* as a potential new prognostic marker for BC.

## 2 Materials and Methods

### 2.1 Dataset Acquisition and Expression Level Quantification

Expression profiles (read counts) of 1,291 The Cancer Genome Atlas (TCGA)-BRCA samples (Ciriello et al., 2015) were downloaded from the Genomic Data Commons portal (https://portal.gdc.cancer.gov). After removing redundant samples and samples with incomplete clinical information, 1194 samples including 1075 tumor, 112 adjacent normal, and 7 metastatic were kept. In this dataset, 111 individuals have normal and tumor paired samples.

In addition, an RNA-seq dataset (SRP324699) was downloaded from The National Center for Biotechnology Information (NCBI) Sequence Read Archive database (https://www.ncbi.nlm.nih.gov/sra), which included 14 paired normal and tumor BC samples. The FASTQ files were aligned with the human reference genome hg38 using TopHat2 (Kim et al., 2013). Reads were counted using htseq-count (Anders et al., 2015) for each gene based on the GENCODE annotation (v38).

Quantile normalization and 10-based log transformations were performed on the raw read counts of the expression data. The following analyses were based on the normalized reads.

Single-cell RNA-seq data were used to characterize the cellular expression specificity in BC. These data were generated from three samples, including a primary tumor, a positive lymph node, and a negative lymph node. The expression matrix was downloaded from the NCBI Gene Expression Omnibus (GEO) database under accession ID (GSE158399) and then processed using the Scanpy pipeline (Wolf et al., 2018). The data matrix from the three samples was merged, cells with expression genes less than 200 were filtered, and filtered genes were expressed in fewer than five cells. We further filtered out the cells that expressed high-level mitochondrial genes (>20%) and cells that expressed more than 4,000 genes. Marker genes were found using rank_genes_groups with parameters “leiden” and “wilcoxon.” Cell types are annotated according to the expression of marker genes (B cells: CD79A, CD79B; T cells: CD3D, IL7R; tumor cells: KRT19; myeloid cells: LYZ; stromal cells: others).

### 2.2 Differential Analysis of Tripartite Motif-Containing Genes

From GENCODE annotation, 115 TRIMs were extracted. The expressions of TRIMs were compared between BC and normal samples. Two-tailed Student’s *t*-test was used to do the comparison. One gene is significantly changed if the absolute log fold change (|logFc|) was >0.58 and the Benjamini–Hochberg false discovery rate–corrected *p*-value <0.05. In addition, paired Student’s t-test was used for paired comparison among the normal tumor samples from the same individuals. In the validation, an RNA-seq dataset (SRP324699) was used, which included 14 paired tumor and adjacent normal samples. The *p*-value cut-off of 0.05 was used.

### 2.3 Correlation Analysis Between Tripartite Motif-Containing Genes and Clinical Features

The clinical information of the TCGA-BRCA samples was downloaded from cBioPortal (https://www.cbioportal.org/) (Gao et al., 2013). Three types of information were considered: molecular subtypes, stages, and age groups. Samples were divided into five molecular subtypes (normal-like, luminal A, luminal B, HER2+, and basal) based on the expression profiles, four stages (I, II, III, IV, and V) according to the American Joint Committee on Cancer (AJCC) pathologic tumor stage, and four age-at-diagnosis groups (<35, 35–50, 50–70, >70).

One-way ANOVA was used to test if there is any difference among the expressions of the candidate gene in different subgroups. A *p*-value after the Bonferroni correction was used to define the significance cut-off (*p* < 0.05/43 = 0.0011). If there is any significant difference in the subgroups, Tukey’s honestly significant difference (HSD) test is further performed to find the significantly different pairs in the subgroups (*p* < 0.05).

### 2.4 Analysis of the Prognostic Value of Tripartite Motif-Containing Genes

The relationship between TRIMs’ expression and the OS of BC patients is analyzed using the Kaplan–Meier curve and log-rank test using Python package lifelines (https://lifelines.readthedocs.io/en/latest/, version 0.26.4; Davidson-Pilon, 2019). Genes with *p* < 0.05 were considered to have prognostic values.

In the validation phase, an independent dataset including 327 individuals was downloaded from the NCBI GEO database (GSE20685). The Kaplan–Meier curve and log-rank test were performed similarly. A statistically significant difference was considered when a log-rank *p* was <0.05.

Univariate and multivariate Cox proportional hazards regression analyses using lifelines were performed on TRIMs and clinicopathological characteristics on the OS of patients. A statistically significant difference was considered when the *p* was <0.05.

### 2.5 Functional Analysis of Gene Set

We conducted a protein–protein interaction network analysis of TRIMs to explore the interactions among them using the STRING database (version 11.5, https://string-db.org/; Szklarczyk et al., 2021), which is a database that collects all publicly available sources of protein–protein interaction. Genes were analyzed using the full STRING network, and the confidence score of 0.4 was used for filtering interactions. In addition to the TRIMs we analyzed, directly interacted genes were added to the network by clicking “More” once from the web interface.

The coexpressed genes are identified using the TCGA-BRCA dataset. The Spearman correlation coefficients were calculated between the target genes and other genes. One gene was considered a coexpressed gene if the |coefficient| was >0.6. As a gene family, we performed the correlation analysis among the selected key genes. The coefficients among the TRIMs were calculated and visualized using a heatmap.

Gene Ontology (GO), Kyoto Encyclopedia of Genes and Genomes (KEGG), pathway and Reactome pathway enrichment analyses were performed using g:Profiler (https://biit.cs.ut.ee/gprofiler; Raudvere et al., 2019) to elucidate the function, biological process, and enrichment pathway of the selected genes. The adjusted enrichment *p*-value was reported by g:Profiler as its default. Biological processes, cellular components, and molecular functions were included in the GO enrichment analysis, while KEGG and Reactome analysis defined the pathways related to the TRIMs.

### 2.6 Statistical Analysis

Statistical analysis and data plotting were performed using Python (version3). The value of 0.05 was the cut-off for the statistical significance unless otherwise specified. The Kaplan–Meier method with a log-rank test was used for survival analysis. Univariable and multivariable analyses were performed using the Cox proportional hazards model. One-way ANOVA was used to compare multiple groups; the HSD test was used to test the difference among subgroups. The Mann–Whitney *U* test was used for the comparison of the TRIM45-high/low groups.

## 3 Results

### 3.1 Differential Analysis of Tripartite Motif-Containing Genes in Breast Cancer

According to the GENCODE, 115 TRIM-related genes were extracted. Of them, 114 TRIMs can be identified from the TCGA-BRCA dataset. There are 110 TRIMs expressed in BC with at least one read detected in one of the samples, while four pseudo-TRIMs (*TRIM60P11Y*, *TRIM60P5Y*, *TRIM60P10Y*, and *TRIM60P9Y*) are not expressed at all in this dataset. Overall, the three most highly expressed genes are *TRIM28*, *TRIM44*, and *TRIM8* in the primary and metastatic tumors and normal tissues.

To explore TRIMs dysregulated in BC, we compared the expression profiles between normal and tumor samples using the TCGA-BRCA dataset (N_tumor_ = 1075 and N_normal_ = 112). The analysis identified 35 significantly differentially expressed genes (DEGs) from 110 TRIMs (p_adjusted_ < 0.05, |logFc| > 0.58). In total, 16 TRIMs (*TRIM15/L2/39-RPP21/71/80P/59/46/11/72/60P18/62/67/3/14/45/28*) are upregulated, while 19 TRIMs (*TRIM61/66/31/63/7/52-AS1/22/68/9/31-AS1/51BP/60P17/2/43B/55/50/51JP/29/75*) are downregulated in BC.

**FIGURE 1:**
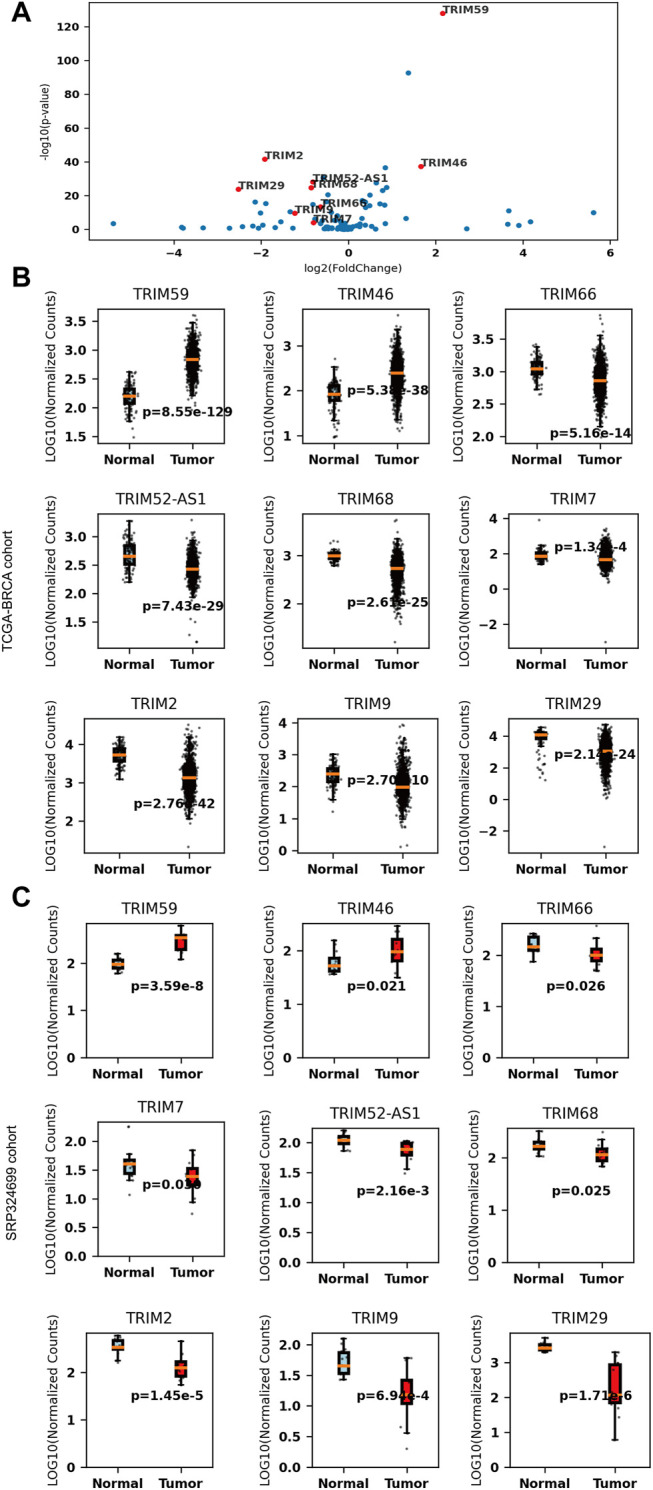
The expression of nine differentially expressed tripartite motif-containing genes (TRIMs) in breast cancer (BC). **(A)** volcano plot of TRIMs in The Cancer Genome Atlas (TCGA)-BRCA cohort **(B)** boxplots of the expressions of upregulated *TRIM59*/*46* and downregulated *TRIM66/52-AS1/68/7/2/9/29* in the TCGA-BRCA cohort (N_Normal_ = 112, N_tumor_ = 1075). **(C)** boxplots of the expressions of upregulated *TRIM59*/*46* and downregulated *TRIM66/52-AS1/68/7/2/9/29* in the SRP324699 cohort (N_Normal_ = 14, N_tumor_ = 14).

To validate these results, we further analyzed another dataset (SRP324699) that included 14 tumor and adjacent normal tissues. Nine of the 35 DEGs are still significant in the independent cohort (*p* < 0.05, |logFc| > 0.58). *TRIM59/46* are consistently upregulated and *TRIM66/52-AS1/68/7/2/9/29* are consistently downregulated in BC. The comparison details are listed in Table 1. These significant TRIMs were highlighted in a volcano plot in Figure 1A, and the boxplots of the expression of the TRIMs are shown in Figure 1B and Figure 1C for TCGA and the validation cohort, respectively.

**TABLE 1:** Differentially expressed TRIMs in BC.

Genes	Official full name	Locus	Discovery dataset (TCGA-BRCA)				Validation dataset (SRP324699)	
			Non-paired		Paired		Fc	*p*
			Fc	*p*	Fc	*p*		
*TRIM59*	tripartite motif containing 59	3q25.33	4.47	8.55e-129	4.98	1.89e-47	3.59	3.59e-08
*TRIM46*	tripartite motif containing 46	1q22	3.17	5.38e-38	2.88	1.67e-18	1.61	0.021
*TRIM66*	tripartite motif containing 66	11p15.4	0.64	5.16e-14	0.57	2.49e-12	0.66	0.026
*TRIM52-AS1*	TRIM52 antisense RNA 1	5q35.3	0.57	7.42e-29	0.55	3.14e-17	0.66	0.022
*TRIM68*	tripartite motif containing 68	11p15.4	0.55	2.61e-25	0.54	1.42e-16	0.64	0.025
*TRIM7*	tripartite motif containing 7	5q35.3	0.57	1.34e-4	0.60	3.62e-04	0.53	0.030
*TRIM2*	tripartite motif containing 2	4q31.3	0.26	2.76e-42	0.28	8.00e-23	0.36	1.45e-05
*TRIM29*	tripartite motif containing 29	11q23.3	0.17	2.14e-24	0.17	2.47e-16	0.041	1.71e-06
*TRIM9*	tripartite motif containing 9	14q22.1	0.43	2.70e-10	0.46	4.52e-06	0.30	6.94e-04

Fc, fold change; *p*, *p*-value; TRIMs, tripartite motif.

Nine genes are still significant when we do a paired comparison among the 111 individuals with normal and tumor samples (*p*_adjusted_ < 0.05, |logFc|>0.58) (Table 1). Six (*TRIM59/46/68/2/9/29*) of the nine TRIMs are also significantly altered in the metastatic tissues when compared with the normal samples (*p*_adjusted_ < 0.05), except TRIM66/52-AS1/7.

As a result, we conclude that *TRIM59*/*46* are upregulated and *TRIM66/52-AS1/68/7/2/9/29* are downregulated in BC. Abnormal expression of these four TRIMs suggested they may be involved in the pathogenesis of BC.

### 3.2 Expression of Tripartite Motif-Containing Genes Correlated to the Molecular Subtypes of Breast Cancer

Next, we analyzed the association between the expression level of TRIMs and clinical features using the TCGA-BRCA dataset. One-way ANOVA was used to test if there was any expression difference of TRIMs in the molecular subtypes (normal-like, luminal A, luminal B, HER2+, and basal), tumor stages (I, II, III, IV, and V), and age-at-diagnosis groups (age groups; <35, 35–50, 50–70, and >70). Tukey’s HSD test was further performed to find significant pairs.

**FIGURE 2:**
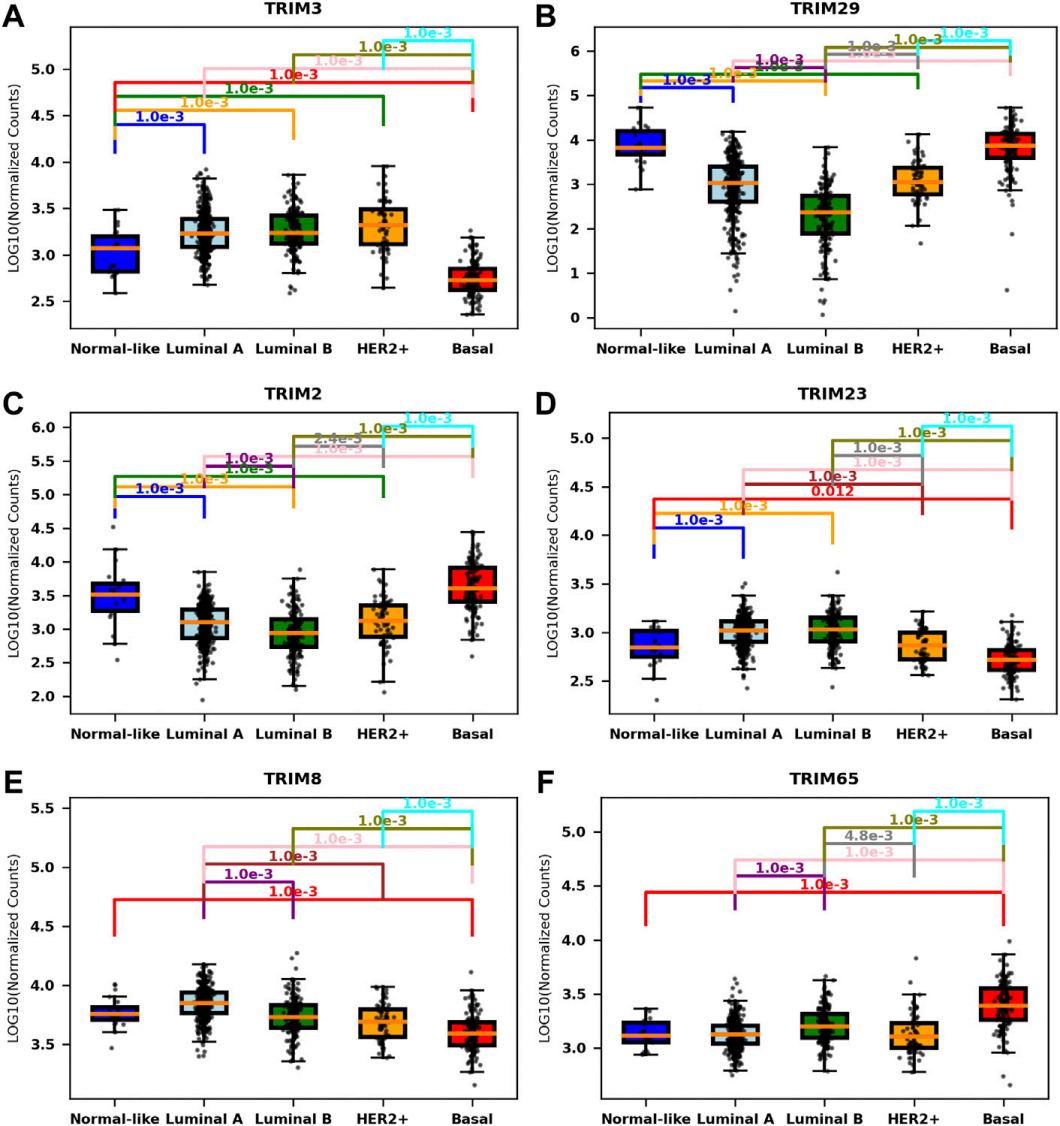
Association of TIRMs’ expression and molecular subtypes in BC. Boxplots of the expressions of the most significant TRIMs in different molecular subtypes. **(A)** *TRIM3*, expressed significantly lower in the normal-like and basal subtypes. **(B)** *TRIM29*, expressed significantly higher in the normal-like and basal subtypes. **(C)** *TRIM2*, expressed significantly higher in the normal-like and basal subtypes. **(D)** *TRIM23*, expressed significantly lower in the basal subtype. **(E)** *TRIM8*, expressed significantly lower in the basal subtype. **(F)** *TRIM65*, expressed significantly higher in the basal subtype.

Overall, approximately 33.67% of TRIMs (37/110) are correlated with molecular subtypes, but only a small portion of TRIMs are associated with age groups (5/110 or 4.55%) or with the clinical stages (1/110 or 1.82%) (*p* < 0.001, F test, Bonferroni correction). The statistic details for the significantly correlated genes are listed in Table 2 (Only six genes with the least *p*-value are listed for molecular subtypes). The most significant one in molecular subtypes is *TRIM3* (*p* = 2.07e−98), which is expressed significantly lower in normal-like and basal BC than in other subtypes (Figure 2A). A different scenario was found for *TRIM29* (*p* = 7.74e−83) and *TRIM2* (*p* = 9.74e−73); they are highly expressed in normal-like and basal BC than in other subtypes (Figure 2B and Figure 2C). The expressions of *TRIM23/TRIM8* are significantly lower in basal than in other subtypes (Figure 2D and Figure 2E), while *TRIM65* is expressed significantly higher in basal than in other subtypes (Figure 2F). The top three genes associated with age groups are *TRIM29* (*p* = 1.38e−05), *TRIM47* (*p* = 2.06e−05), and *TRIM24* (4.80e−05).

**TABLE 2:** Association of TRIMs’ expression and breast cancer clinical features.

Characteristics	Genes	F test		Significant pairs (Tukey’s honestly significant difference test)^a^
		F-value	*p*	
Molecular subtypes^b^	*TRIM3*	155.70	2.07e-98	G1 vs. (G2, G3, G3, G4, G5); G2 vs. G5; G3 vs. G5; G4 vs. G5
	*TRIM29*	125.05	7.74e-83	G1 vs. (G2, G3, G4); G2 vs. (G3, G5); G3 vs. (G4, G5); G4 vs. G5
	*TRIM2*	106.59	9.74e-73	G1 vs. (G2, G3, G4); G2 vs. (G3, G5); G3 vs. (G4, G5); G4 vs. G5
	*TRIM23*	96.40	6.65e-60	G1 vs. (G2, G3, G5); G2 vs. (G4, G5); G3 vs. (G4, G5); G4 vs. G5
	*TRIM8*	84.50	7.78e-60	G1 vs. G5; G2 vs. (G3, G4, G5); G3 vs. G5; G4 vs. G5
	*TRIM65*	74.82	7.27e-54	G1 vs. G5; G2 vs. (G3, G5); G3 vs. (G4, G5); G4 vs. G5
Age-at-diagnosis groups	*TRIM29*	8.23	1.38e-05	G2 vs. G3; G2 vs. G4
	*TIRM47*	8.22	2.06e-05	G1 vs. G3; G1 vs. G4; G2 vs. G3; G2 vs. G4
	*TRIM24*	7.62	4.80e-05	G1 vs. G2; G1 vs. G3; G1 vs. G4; G2 vs. G4
	*TRIM65*	6.57	2.13e-04	G2 vs. G3; G2 vs. G4
	*TRIM52*	5.70	7.20e-04	G2 vs. G3; G2 vs. G4
Stage (I, II, III, IV, and V)	*TRIM3*	6.37	0.00028	G2 vs. G3; G2 vs. G4

Tested using one-way ANOVA.

a Tumor subtypes: G1: normal-like, G2: luminal A, G3: luminal B, G4: HER2+, G5: basal; age-at-diagnosis groups: G1: <35, G2: 35–50, G3: 50–70, G4: >70; stages: G1: stage I, G2: stage II, G3: stage III, G4: stage IV and V.

b Only six top associated TRIMs were shown in the table.

In conclusion, many TRIMs are significantly correlated with molecular subtypes, but not with tumor stages or patient age at diagnosis.

### 3.3 Tripartite Motif-Containing Genes Associated With Prognosis of Breast Cancer

We next investigated the prognosis value of TRIMs’ expression in BC. The relationship between the expression of TRIMs and the OS of BC patients was analyzed using the Kaplan–Meier curve and log-rank test using the TCGA-BRCA dataset. The analysis revealed that the expressions of 37 TRIMs are significantly associated with the OS of BC (*p* < 0.05, log-rank test).

**FIGURE 3:**
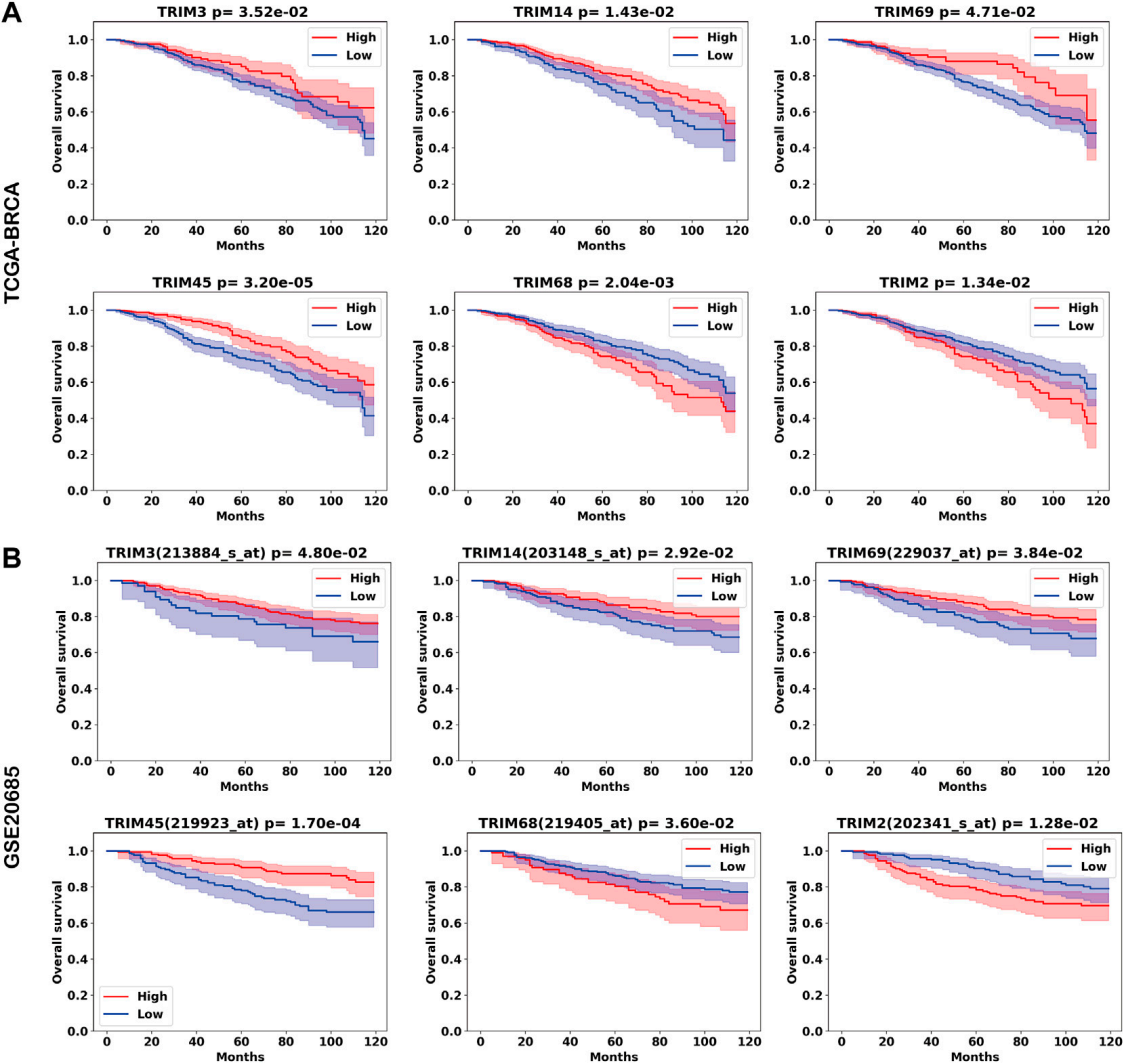
Tripartite motif (TRIMs) associated with the prognosis of BC. The Kaplan–Meier curves for genes *TRIM3/14/69/45/68/2* in **(A)** TCGA-BRCA cohort and **(B)** the GSE20685 cohort.

To validate these results, we performed Kaplan–Meier analysis using an independent dataset. Six TRIMs are validated from 24 genes from the TCGA-BRCA cohort. Higher expressions of *TRIM3*, *TRIM14*, *TRIM69*, and *TRIM45* are associated with a favorable OS, while higher expressions of *TRIM68* and *TRIM2* are associated with a worse OS in BC. The Kaplan–Meier curves for the six genes are shown in Figures 3A,B for the TCGA-BRCA and GSE20685 cohorts.

Of the nine DEGs, *TRIM2* and *TRIM68* are significantly associated with worse OS in both the TCGA-BRCA cohort and the validation cohort. Moreover, while *TRIM2* and *TRIM68* are downregulated in BC, higher expressions of *TRIM2* and *TRIM68* are associated with worse OS in BC. Of the seven upregulated genes, there is no significant gene identified.

In the previous analysis, TRIMs’ expressions are associated with BC subtypes. Of the six TRIMs (*TRIM3*/*14/69/45/2/68*) identified from Kaplan–Meier analysis, 5/6 (83.3%) were associated with molecular subtypes except for *TRIM14*; *TRIM3* was associated with stages. To investigate the independent role of TRIMs, we applied a univariate and multivariate Cox proportional hazards model to the expression data. In the univariant analysis, ages, luminal A subtype, HER2+ subtype, stages, and the expression of *TRIM69*/*45* are associated with the OS of BC (Table 3). In the multivariate analysis, high expression of *TRIM45* demonstrates an independent role (from molecular subtypes, ages, and stages) with a better prognosis in BC (Table 3).

**TABLE 3:** Univariate and multivariate Cox proportional hazards analyses for TRIMs, ages, stages, and molecular subtypes in breast cancer.

Characteristics^a^		Univariate analysis		Multivariate analysis	
		Hazard ratio (95% CI)	*p*	Hazard ratio (95% CI)	*p*
Age		1.03 (1.02-1.05)	**<0.005**	1.04 (1.02-1.05)	**<0.005**
Molecular subtypes	Lumina A	0.66 (0.46-0.94)	**0.02**		
	Lumina B	1.43 (0.93-2.21)	0.10		
	Her2+	2.29 (1.31-4.01)	**<0.005**	1.92 (1.04-3.55)	**0.04**
	Basal	0.89 (0.54-1.45)	0.63		-
	Normal-like	0.99 (0.36-2.67)	0.98		-
AJCC stages	I	0.37 (0.21-0.68)	**<0.005**	0.19 (0.10-0.36)	**<0.005**
	II	0.66 (0.46-0.95)	**0.03**	0.38 (0.26-0.56)	**<0.005**
	III	2.12 (1.44-3.12)	**<0.005**	1.94 (1.30-2.88)	**<0.005**
	IV and V	3.88 (2.24-6.69)	**<0.005**		
*TRIM3*		1.40 (0.76-2.60)	0.28	-	-
*TRIM14*		0.73 (0.32-1.67)	0.46	-	-
*TRIM69*		0.36 (0.13-0.96)	**0.04**	-	-
*TRIM45*		0.40 (0.20-0.80)	**0.01**	0.47 (0.23-0.99)	**0.05**
*TRIM68*		1.18 (0.59-2.35)	0.65	-	-
*TRIM2*		1.08 (0.68-1.71)	0.74	-	-

a Age, numeric; molecular subtypes: (five) categories were encoded into binary categories; AJCC stages: (four) categories were encoded into binary categories.

AJCC, American Joint Committee on Cancer; CI, confidence interval;.*p*, *p*-value; TRIMs, tripartite motif-containing genes.

The bold value means “significant *p* value”. Also need bold other 3 *p* values less than 0.05.

Based on the analysis, the expression of *TRIM45* may be used as a prognosis marker for BC, while the prognosis value of *TRIM3/14/69/68/2* may need to be further investigated.

### 3.4 Expression Specificity of Tripartite Motif-Containing Genes in Cell Types

**FIGURE 4:**
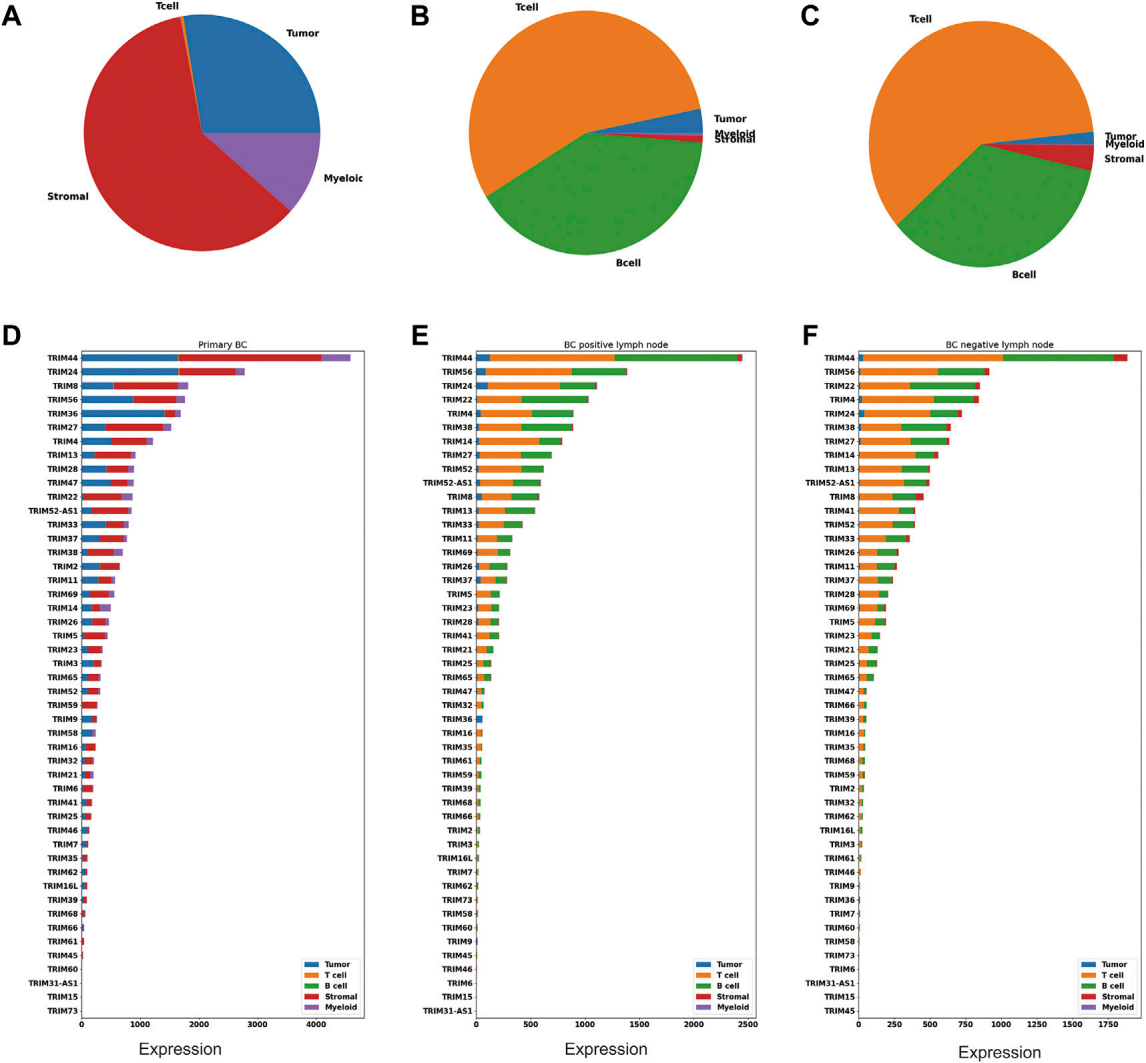
Cell-type expression specificity of TRIMs in BC. Pie plots showing the percentage of the tumor, myeloid, stromal, B, and T cells in **(A)** the primary tumor, **(B)** the positive lymph node, and **(C)** the negative lymph node using GSE158399. Bar plots showing the expression of TRIMs in different cell types for **(D)** the primary tumor, **(E)** the positive lymph node, and **(F)** the negative lymph node.

BC is shown to have high cellular heterogeneity; next, we profiled a single-cell dataset (GSE158399) to investigate if there is any cell-type expression specificity in TRIMs. This piece of data includes three samples: primary BC, positive lymph node, and negative lymph node. Based on cell markers, these cells were annotated into five categories: tumor cells, T cells, B cells, stromal cells, and myeloid cells. The cell number in each cell type were counted and visualized using a pie plot (Figurea 4A–C). There are 48 TRIMs expressed in this dataset. The expression levels of these genes are summarized according to cell types and sample types (Figures 4D–F). We found that most TRIMs are expressed in the tumor and stromal cells in the primary BC, and the expression in the nontumor cell types are accounting for a large proportion of the total expression. Some genes (*TRIM22/TRIM38/TRIM5/TRIM59*) are expressed lower in tumor cells than in other cell types (Figures 4D–F). In addition, numerous TRIMs are highly expressed in the T cells and B cells in the BC lymph nodes (negative and positive), which may correspond to their important role in immunity.

### 3.5 Interaction, Co-expression, and Functional Analysis of Tripartite Motif-Containing Genes

**FIGURE 5:**
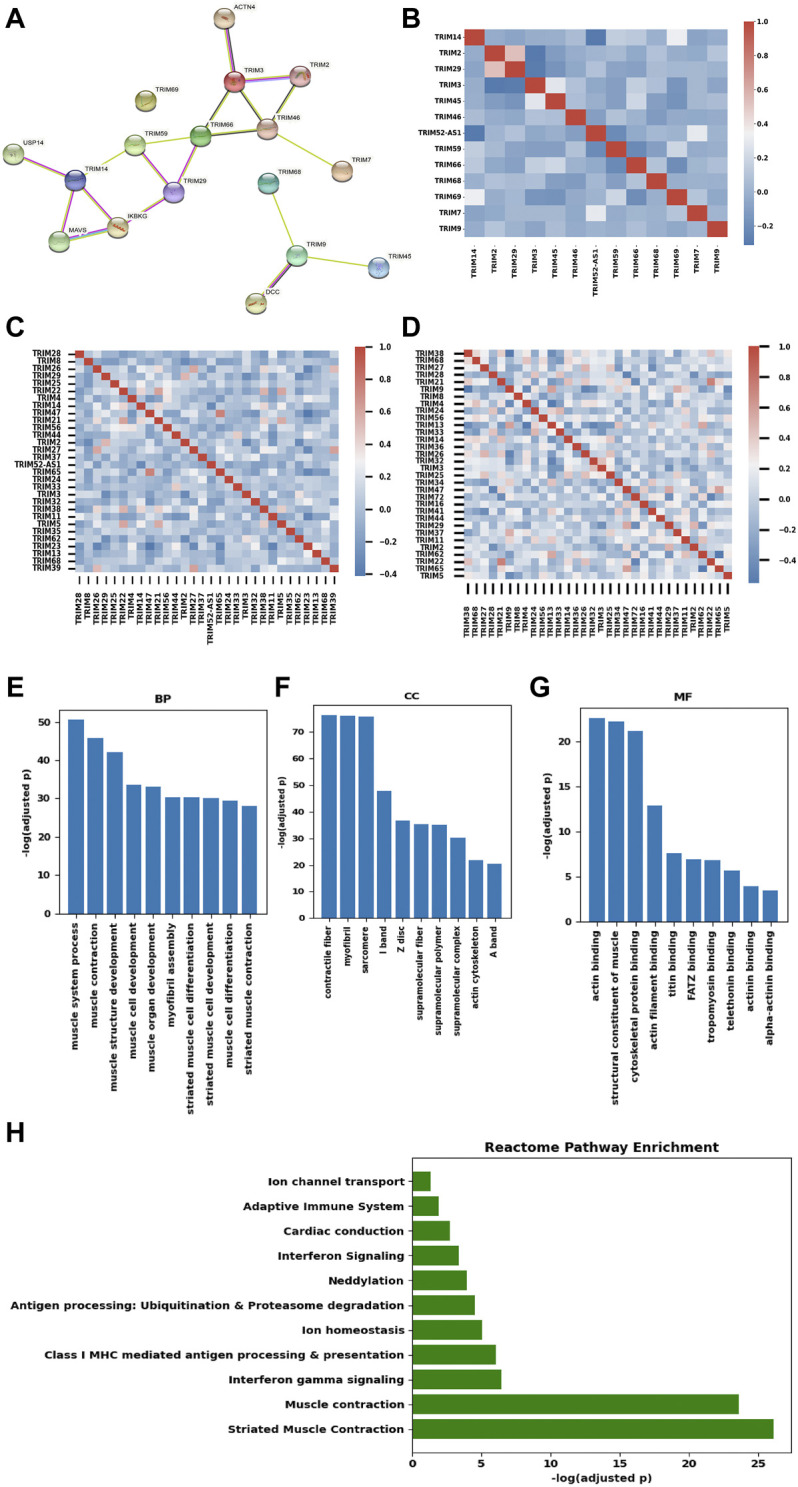
Interaction, co-expression, and functional analysis of TRIMs. **(A)** the protein–protein interaction network of TRIMs generated using STRING. **(B)** heatmap of the Pearson’s correlation efficiency of the 13 candidate TRIMs. **(C)** heatmap of the correlation efficiency of top 30 expressed TRIMs in BC. **(D)** heatmap of the correlation efficiency of 32 TRIMs with protein expression in TCGA-BRCA cohort. **(E)** bar plot of the enriched biological process (BP) items. **(F)** bar plot of the enriched cellular component (CC) items. **(G)** bar plot of the enriched molecular function (MF) items. **(H)** Bar plot of the enriched Reactome pathways.

The interactions of TRIMs were investigated using the STRING database. Nine DEGs (*TRIM59*/*46/66/52-AS1/68/7/2/9/29*) and six genes (*TRIM3*/*14*/*69/45/68*/*2*) in the Kaplan–Meier analysis were searched in the STRING database (https://string-db.org/). TRIM52-AS1 is a non-coding RNA, and the other 12 genes and 5 directly interacted non-TRIM genes formed three disconnected networks (Figure 5A). TRIM69 itself, which did not interact with any other genes, formed an isolated network. One small network was formed by three TRIMs (TRIM45, TRIM68, TRIM9) and a non-TRIM gene DCC netrin 1 receptor (DCC). DCC is considered a tumor suppressor; downregulation of TRIM9 and TRIM68 in BC may lead to changes in BC, although we did not see the correlation in the mRNA level (Spearman coefficient r = 0.022, 0.043). The third network includes eight TRIMs (TRIM2/7/46/3/66/59/29/14) that interacted with four non-TRIM proteins: actinin alpha 4 (ACTN4), inhibitor of nuclear factor kappa B kinase regulatory subunit gamma (IKBKG), mitochondrial antiviral signaling protein (MAVS), and ubiquitin-specific peptidase 14 (USP14). TRIM14 interacted with USP14, MAVS, and IKBKG, while TRIM14 is downregulated in BC, although we also did not see the correlations in the mRNA level (r = 0.10, 0.026, 0.052). In addition, we also did observe the correlation between *TRIM3* and *ACTN4* in the mRNA level (r = -0.075).

To investigate the possible function of the 13 TRIMs, their co-expression genes were calculated using Spearman correlation in the TCGA-BRCA dataset. The correlations among these TRIMs were limited (Figure 5B). We further checked the correlations in the top 30 expressed TRIMs in BC, and the heatmap suggests the low correlation in this family (Figure 5C). In addition, the correlations of 32 TRIMs with protein expression information in the TCGA-BRCA cohort are shown in Figure 5D. In conclusion, low expression correlations were found among TRIMs. Including the 13 TRIMs, there were 405 genes coexpressed with the targeted TRIMs (r > 0.6). No gene was correlated with *TRIM68/9/46/52-AS1/14/45/59* under the threshold r > 0.6. GO and KEGG/Reactome pathway enrichment was performed based on the coexpressed genes using the Gprofiler. In the biological process (BP) category, the three most enriched items are the muscle system process, muscle contraction, and muscle structure development (Figure 5E). Contractile fiber, myofibril, and sarcomere are the three most enriched cellular component (CC) items (Figure 5F). The three most significant GO items in the molecular function (MF) category are the actin binding/structural constituent of muscle/cytoskeletal protein binding (Figure 5G). The KEGG pathway enrichment analysis indicated that these genes were enriched in the cardiac muscle contraction pathway. Reactome pathway enrichment suggested that these genes were enriched in several immune-related pathways (interferon-gamma signaling/antigen processing and presentation/adaptive immune system) beside the pathways related to the muscle contraction (Figure 5H).

**FIGURE 6:**
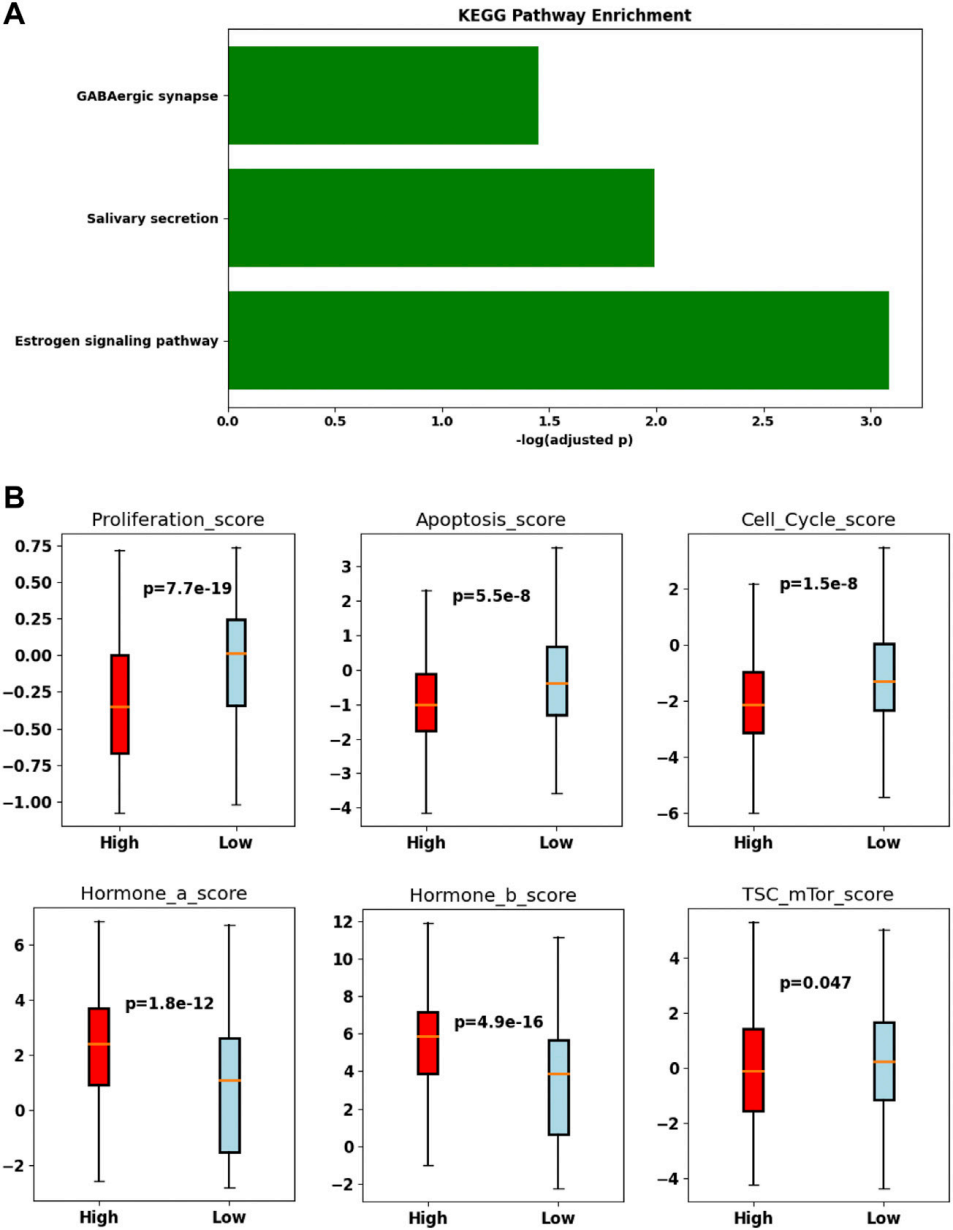
Difference in the TRIM45-group and the TRIM45-low group. **(A)** bar plot of the enriched Kyoto Encyclopedia of Genes and Genomes (KEGG) pathway. **(B)** Boxplot of proliferation score, apoptosis score, cell cycle score, hormone a score, hormone b score, TSC mTor score.

Since we did not see any gene coexpressed with *TRIM45*, we divided the samples from the TCGA-BRCA cohort into TRIM45-high and TRIM45-low groups according to the median expression of *TRIM45*. A comparison of the expression profile between the two groups identified 961 significantly DEGs. The estrogen signaling pathway was found enriched in these genes by KEGG pathway enrichment analysis (Figure 6A). In addition, we found that the TRIM45-high group has a significantly lower proliferation score, apoptosis score, cell cycle score, and TSC mTor score and a significantly higher hormone a score and hormone b score (Mann–Whitney *U* test, *p* < 0.05) (Figure 6B). The results suggested that TRIM45 may be involved in the hormone response and other cellular functions like proliferation. The TRIM45-high group with a lower proliferation score, cell cycle score, and mTor score and higher hormone a/b score may partially explain the better OS of this group.

## 4 Discussion

The TRIM family has many members, some of which are reported to contribute to the pathogenicity of BC recently. In this study, we integrated several public datasets to explore the expression profile of all TRIMs in BC. Nine DEGs (*TRIM59*/*46*/*66/52-AS1/68/7/2/9/29*) were identified and validated using two independent datasets. In addition, we found that six TRIMs have prognosis values using two independent datasets. Higher *TRIM3/14/69/45* expression and lower *TRIM68/2* expression were associated with favorable OS in BC patients. The expression of TRIM45 is an independent prognosis factor from patient age, tumor stages, and molecular subtypes.

The alterations of the expression levels of the nine genes may suggest their important role in BC progression. *TRIM59* overexpression can promote BC progression (Liu et al., 2018), while genetic depletion of this gene suppresses BC metastasis (Tan et al., 2019). Knockdown of TRIM66 in the BC cell line can suppress proliferation (Zhang et al., 2021). *TRIM29* is functional as a tumor suppressor in BC (Ai et al., 2014). The functions of *TRIM2*/*TRIM46/TRIM68/TRIM9/TRIM52-AS1/TRIM7* are not clear in BC yet. In other cancers, *TRIM7* can suppress hepatocellular carcinoma progression (Zhu et al., 2020), while an oncogenic function was reported in colorectal cancer (Cao et al., 2019) and pancreatic cancer (Sun et al., 2020) for *TRIM2* and in hepatocellular carcinoma for *TRIM52-AS1* (Zhou et al., 2021). Of the nine genes, *TRIM2* is downregulated in BC, and it is also found to be associated with a better OS in BC.

Six genes were identified to have prognostic values in BC. Higher *TRIM3/14/69/45* expression and lower *TRIM68/2* expression are associated with favorable OS in BC. The three TRIMs [*TRIM21* (Zhou et al., 2018), *TRIM44* (Kawabata et al., 2017), and *TRIM13* (Chen et al., 2019)] reported to have prognostic values in BC were not validated in our analysis. *TRIM14* was reported to promote BC proliferation (Hu et al., 2019), while the role of *TRIM3* is somewhat contradictory in BC (Liu et al., 2014; Wang et al., 2020). Depletion of TRIM68 can inhibit colorectal cancer cell proliferation (Tan et al., 2017). Limited information is found for *TRIM69*.

*TRIM45* is the only gene from the TRIM family with an independent effect on the OS of BC using multivariate Cox regression analysis. Higher expression of TRIM45 was found in the luminal A and luminal B subtypes than in other molecular subtypes. Higher expression was also found in the stage I group than in the stage III group. In addition, we found that the TRIM45-high group was with higher hormone scores and lower proliferation/cell cycle scores. These may help explain the better OS in the higher group. The single-cell RNA-seq data demonstrated that it is expressed in nontumor cells. It is reported as a tumor suppressor gene in glioma (Zhang et al., 2017), but few studies have reported it can be found in BC. Further functional study of *TIRM45* in hormone response may help clarify its role in BC.

Correlation analysis demonstrates that TRIMs have low expression correlations with each other and with other genes in BC. The low correlations may suggest that they prefer working alone or may be because they function as an ubiquitin ligase, which involves protein-level modifications. Pathway enrichment analysis of the co-expression genes indicates their functions are related to muscle contraction and immunity. The dysregulation of muscle-related pathway in BC was reported before (Bohlen et al., 2018). The single-cell-level expression of TRIMs revealed that most TRIMs are also expressed in the immune and stromal cells, suggesting that they have an important role in the BC microenvironment. Functional studies of their role in the immune or stromal cells may help elucidate their roles in BC progression.

In conclusion, through the bioinformatics analysis, we report nine DEGs and six candidate genes linked to BC prognosis in the TRIM family. Some TRIMs (*TRIM45/46/2/69*) are not well-studied in BC, and *TRIM45* may serve as a novel biomarker for BC.

## Data Availability

The original contributions presented in the study are included in the article, and further inquiries can be directed to the corresponding author.
